# The Application of SILAC Mouse in Human Body Fluid Proteomics Analysis Reveals Protein Patterns Associated with IgA Nephropathy

**DOI:** 10.1155/2013/275390

**Published:** 2013-05-15

**Authors:** Shilin Zhao, Rongxia Li, Xiaofan Cai, Wanjia Chen, Qingrun Li, Tao Xing, Wenjie Zhu, Y. Eugene Chen, Rong Zeng, Yueyi Deng

**Affiliations:** ^1^Key Laboratory of Systems Biology, Institute of Biochemistry and Cell Biology, Shanghai Institutes for Biological Science, Chinese Academy of Sciences, Shanghai 200031, China; ^2^Department of Nephrology, Longhua Hospital, Shanghai University of Traditional Chinese Medicine, 725 Wanping Road, Shanghai 200032, China; ^3^Cardiovascular Centre, Department of Internal Medicine, University of Michigan Medical Centre, Ann Arbor, MI 48109, USA

## Abstract

Body fluid proteome is the most informative proteome from a medical viewpoint. But the lack of accurate quantitation method for complicated body fluid limited its application in disease research and biomarker discovery. To address this problem, we introduced a novel strategy, in which SILAC-labeled mouse serum was used as internal standard for human serum and urine proteome analysis. The SILAC-labeled mouse serum was mixed with human serum and urine, and multidimensional separation coupled with tandem mass spectrometry (IEF-LC-MS/MS) analysis was performed. The shared peptides between two species were quantified by their SILAC pairs, and the human-only peptides were quantified by mouse peptides with coelution. The comparison for the results from two replicate experiments indicated the high repeatability of our strategy. Then the urine from Immunoglobulin A nephropathy patients treated and untreated was compared by this quantitation strategy. Fifty-three peptides were found to be significantly changed between two groups, including both known diagnostic markers for IgAN and novel candidates, such as Complement C3, Albumin, VDBP, ApoA,1 and IGFBP7. In conclusion, we have developed a practical and accurate quantitation strategy for comparison of complicated human body fluid proteome. The results from such strategy could provide potential disease-related biomarkers for evaluation of treatment.

## 1. Introduction

Body fluid proteome generally is the most informative proteome from a medical viewpoint. Almost all tissues in the body communicate with body fluid and release their contents into it, especially upon damage or death [1]. This complicated matrix is believed to reflect the function changes within the body. Therefore, it is promising to identify novel, highly sensitive, and specific disease biomarkers from body fluid proteomics research [2]. With the significant advances in proteomic methods and instrumentation, current methods are sufficient to allow the identification of proteins directly in body fluid across no less than 7 orders of magnitude of protein abundance [3]. However, because of the high complexity and high dynamic range of protein concentrations in such samples, discovery of disease biomarkers from body fluid proteome remains extremely challenging [4, 5].

Multiple analytical approaches have been developed based on separation of proteins in-gel (2DE, DIGE) or gel-free platform utilizing various methods including labeling (ICAT, ^18^O-labeling, iTRAQ) and label-free strategy. Both of these methods were widely used in quantitative proteomics analysis but also had some limitations. Label-free quantification was quite simple and straightforward, but the precision and accuracy were limited [6]. ^18^O-labeling leaded to variability in peptide spacing, and the mass offset of 2 Da was not sufficient to optimally separate the isotopic envelopes for differential quantification. iTRAQ technology provided an extensive quantification method that could be used in almost all kinds of samples with great utility. But because of the additional multiple steps of iTRAQ method, such as sample preparation and efficiency of chemical tagging, there was also some variability in this method. Besides, the reporter ions in MS/MS spectrum would influence the peptide identification [7].

Stable-isotope labeling by amino acids in cell culture (SILAC) method provided a comprehensive, robust, and accurate solution for quantitative proteomics analysis. But it is thought to be unsuitable for analyzing tissue and body fluid samples. Hence, a lot of other metabolic labeling methods were introduced in an alternative way such as protein standard absolute quantification (PSAQ), stable isotope-labeled proteome (SILAP), and secretome-derived isotopic tag (SDIT) strategies. However, PSAQ uses full-length isotope-labeled proteins as isotope-dilution standards for MS-based quantification of target proteins in complex matrices. It was difficult for the accurate quantitation of large numbers of serum proteins. In addition, it seems more appropriate for the combination of PSAQ and SRM (PSAQ-SRM) to allow highly accurate biomarker quantitation in serum samples. Serum mixed with SILAC-labeled secretome of culture cells (SILAP and SDIT) was easy to manipulate and quantify large numbers of serum proteins. However, serum contains all tissue proteomes as subsets. It was not adequate to use one or several kinds SILAC labeled secretome of cultured cells as standards in quantitative serum proteomic studies.

In this paper, we have proved that the serum from SILAC mouse could also be used as internal standards to quantify human serum and urine. With this novel strategy, we performed the quantitative proteomics analysis by extensive multidimensional separation coupled with tandem mass spectrometry (IEF-LC-MS/MS) for the urine from Immunoglobulin A nephropathy (IgAN) patients treated and untreated. As the quantification standards display the same biochemical features as urinary samples, they can be spiked into the samples at early stages of the analytical process, which can avoid differences that yielded in digestion and nonspecific losses suffered during extensive sample processing between internal standard SILAC mouse and human samples. In conclusion, our research provided a comprehensive and accurate solution for human body fluid analysis.

## 2. Materials and Methods

### 2.1. Materials

Urea, tris, sodium dodecyl sulfate (SDS), dithiothreitol (DTT), ammonium bicarbonate (NH_4_HCO_3_), and iodoacetamide (IAA) were purchased from Bio-Bad (Hercules, CA). The Lys-C enzyme was purchased from Wako (Osaka, Japan). Acetonitrile (HPLC grade) was obtained from Merck (Darmstadt, Germany). Formic acid was obtained from Aldrich (Milwaukee, WI, USA). All the water used in the experiments was prepared using a Milli-Q system (Millipore, Bedford, MA, USA). The ^13^C_6_-lysine labeled mouse serum (MT-LYSC6-MSE) was purchased from Cambridge Isotope Laboratories, Inc.

### 2.2. Sample Collection and Preparation

Immediately after collection, fasting blood samples of healthy subjects were allowed to clot at room temperature for four hours, and the serum was collected and centrifugated at 3000 rpm/min for 15 min. Informed consent was obtained from each person in written format and approved by Longhua Hospital, Shanghai University of Traditional Chinese Medicine Review Committee.

The 24 h urine samples were collected from six healthy subjects. Informed consent was obtained from each person in written format and approved by Longhua Hospital, Shanghai University of Traditional Chinese Medicine Review Committee. Volume of 200 ml urine from each person was concentrated by the acetone precipitation approach [8] with some modification. All centrifugation work was performed at 4°C. First, the samples were centrifuged at 3000 g for 10 min to pellet exfoliated cells and residues. The supernatants were centrifuged at 16,000 g for 30 min to remove cell debris and residues. Then, the urine samples were precipitated by adding chilled acetone to 20% w/v and incubating the samples for 10 min at 4°C. Precipitates were sedimented at 16,000 g for 30 min at 4°C, and pellets were washed twice with neat acetone at 4°C with residual acetone removed by air drying. Dried pellets were resuspended in 3 ml of 50 mM NH_4_HCO_3_ buffer. The protein concentration of urine samples was determined by Bradford assay on a Microplate Reader (Bio-Rad, Model 680). Six normal urine samples were pooled together. The concentrate was frozen at −80°C until use.

The urine from IgAN patients with and without glucocorticosteroid treatment was collected and prepared as mentioned earlier for urine from healthy persons [9]. The patients of glucocorticosteroid treatment group were given one pack Bid (twice per day) only for 24 weeks (Bushen tongluo Decoction or Zibu ganshen Decoction). The dosage of glucocorticosteroid is 0.5–1 mg/kg.d. The glucocorticosteroid drugs were reduced regularly.

### 2.3. In-Solution Lys-C Digestion and OFFGEL Fractionation

The sample digestion was processed by FASP procedure as described previously [10]. Briefly, 150 *μ*g human sample was mixed with 150 *μ*g SILAC-labeled mouse serum sample and then transferred to a 10K filter and centrifuged at 10,000 g for 20 min at 20°C. so, 200 *μ*L UA buffer (8 mol/L urea and 0.1 mol/L Tris-HCl, pH = 8.5) was added, and the sample was centrifuged at 10,000 g for 20 min again. This step was repeated once. The concentration was then mixed with 100 *μ*L of 50 mmol/L IAA in UA buffer and incubated for an additional 40 min at room temperature in the dark. Next, the IAA was removed by centrifugation at 10,000 g for 20 min. Following two rounds of dilution in 200 *μ*L of UA buffer and centrifugation, 200 *μ*L of 50 mmol/L NH_4_HCO_3_ was added to the sample, which was then centrifuged at 10,000 g for 20 min. This step was repeated twice. Finally, 100 *μ*L of 50 mmol/L NH_4_HCO_3_ and Lys-C (1 : 25, enzyme to protein) was added, and the mixture was incubated at 37°C for 20 h. The digested peptide mixtures were collected for further analysis.

The p*I*-based peptide OFFGEL separation was performed as previously reported [10]. The 3100 OFFGEL Fractionator and the OFFGEL Kit pH 3–10 (both Agilent Technologies) with a 12-well setup were used according to the protocol of the supplier. The lyophilized samples were dissolved in focusing buffer to a final volume of 1.8 mL, and 150 *μ*L of sample was loaded in each well. Electrofocusing of the peptides was performed at 20°C and 50 *μ*A until the 100 kVh level was reached. After focusing, the rinses in each well were pooled with their corresponding peptide fraction. All fractions were evaporated by centrifugation under vacuum and maintained at −20°C. Prior to MS analysis, the samples were desalted onto an Empore C18 47-mm Disk. Immediately prior to nano-LC, the fractions were suspended in 20 *μ*L of H_2_O containing 0.1% (v/v) TFA.

### 2.4. 1D Nano-LC-MS/MS Analysis

A Surveyor liquid chromatography system (Thermo Finnigan, San Jose, CA, USA), consisting of degasser, MS Pump, and auto sampler, equipped with an analytical C18 column (RP, 75 *µ*m × 150 mm, Column Technology Inc., CA, USA) was used. The HPLC solvents used were 0.1% formic acid (v/v) aqueous (A) and 0.1% formic acid (v/v) acetonitrile (B). The reversed-phase gradient was from 2% to 40% mobile phase B in 180 min at 120 *µ*L/min flow rate before the split and 250 nL/min after the split. A linear ion trap/Orbitrap (LTQ-Orbitrap) hybrid mass spectrometer (ThermoFinnigan, San Jose, CA, USA) equipped with an NSI nanospray source was used to for MS/MS experiment with ion transfer capillary of 200°C and NSI voltage of 1.80 kv. Normalized collision energy was 35.0. The mass spectrometer was set that one full MS scan (m/z 400–1800) was acquired in the Orbitrap parallel ten MS/MS scans in the linear ion trap on the ten most intense ions from the full MS spectrum with the following Dynamic Exclusion settings: repeat count 2, repeat duration 30 seconds, and exclusion duration 90 seconds. The resolving power of the Orbitrap mass analyzer was set at 60,000 for the precursor ion scans (m/Δm 50% at m/z 400). The m/z (445.120025) was used as an internal lock mass and calibrant ions in the full MS scan.

### 2.5. Data Processing and Quantification

All raw mass spectrometric data were analyzed with the MaxQuant software (version 1.3.0.5 [11]. A false discovery rate (FDR) of 0.01 for proteins and peptides and a minimum peptide length of 7 amino acids are required. MS/MS spectra were searched by the Andromeda search engine incorporated in the MaxQuant software [12] against a combined IPI database (IPI HUMAN version 3.87, containing 91,491 entries and IPI MOUSE version 3.87, containing 59,546 entries) and concatenated with the reversed versions of all sequences. Lys-C digestion (Lys-C/P) was chosen as enzyme specificity. Cysteine carbamidomethylation was selected as a fixed modification, while protein N-terminal acetylation and methionine oxidation were selected as variable modifications. Maximally one missed cleavage was allowed. Initial mass deviation for the precursor ion was up to 7 ppm, and maximum allowed mass deviation for fragment ions was 0.5 Da.

In quantification, Lys6 in MaxQuant software was selected as heavy internal standard to get the intensity for heavy and light peptide and the SILAC ratio. The peptides shared between human and mouse were quantified by their SILAC pairs, and the human-only peptides were quantified by mouse peptides with nearest retention time.

### 2.6. Bioinformatics Analysis

All statistical analyses were conducted in the R environment (http://www.r-project.org/). The quantile normalization [13] was performed to normalize the quantitation results. The seqKNN method [14] was used for imputation of the missing values. The linear models in R package limma were applied to find significant differently expressed proteins [15].

## 3. Results and Discussion

### 3.1. The Experiment Design

SILAC method provided a comprehensive, robust, and accurate solution for quantitative proteomics analysis. And the development of SILAC mouse expanded its application into tissue and body fluid samples [16, 17]. The high similarity between samples and internal standard made SILAC mouse a valuable tool and facilitated proteomics research in many aspects. But currently quantitative researches for human body fluid proteome were still using label-free or iTRAQ methods. SILAC mouse has never been considered to be used in human subjects.

**Figure 1:**
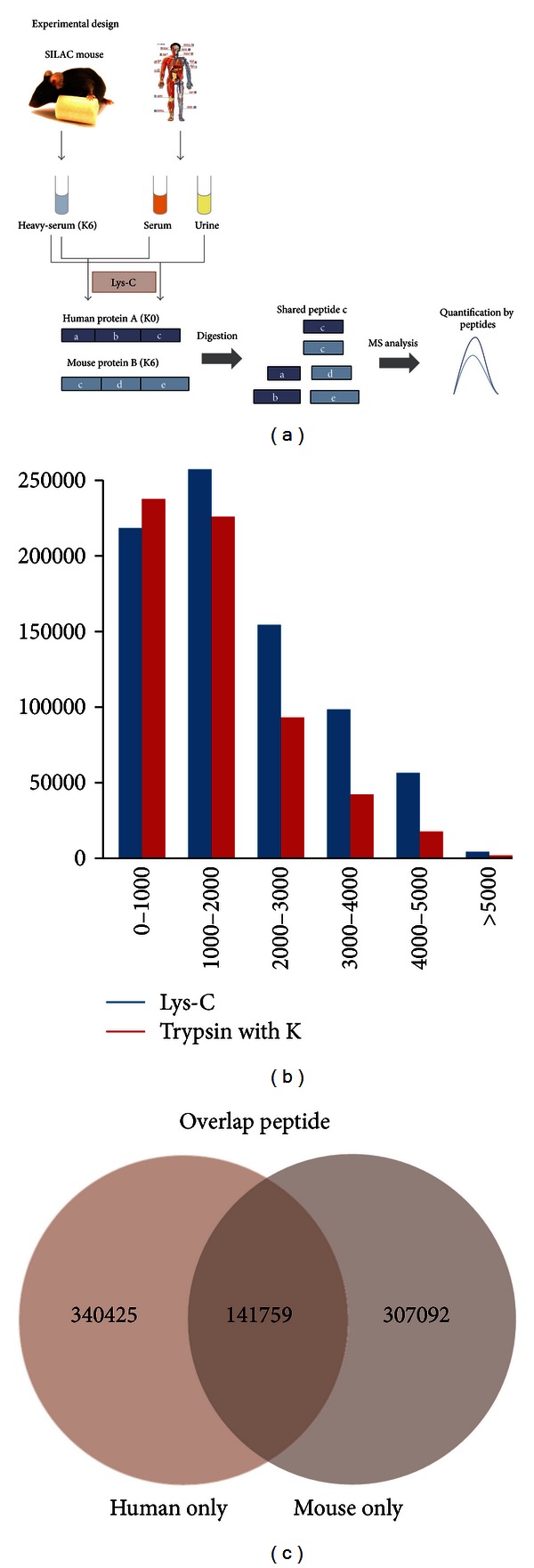
The experiment design and theoretic digestion result. (a) The serum of SILAC mouse was mixed with serum and urine from human, and then enzyme digestion was performed. The shared digested peptides between human and mouse could be selected as SILAC pair and quantified. (b) The comparison of molecular weight distribution for the theoretic digestion peptides of trypsin and Lys-C. (c) The comparison of Lys-C theoretic digestion peptides in human and mouse database.

The major obstacle for the application of SILAC mouse in human samples was the different protein sequence between the two species. But as a necessary step of protein digestion in bottom-up proteomics, the compared results were in fact not protein sequences but digested peptide sequences, which increased the possibility for human sequence to find corresponding SILAC pair in labeled mouse internal standard. Even the proteins with different sequence between human and mouse may have consistent digested peptides and could be quantified. Hence, we proposed a novel workflow, in which the SILAC-labeled mouse serum was used as internal standard to quantify human body fluid (Figure 1(a)).

As the serum of SILAC mouse was labeled by lysine, the lysine-containing peptides from human samples and mouse internal standard could be distinguished in MS analysis, and the peptides without lysine were undistinguishable and helpless for quantitative analysis. To find the enzyme with better performance for such kind of experiment design, trypsin and Lys-C theoretic digestions for human IPI proteome database were performed and compared. As a result, Lys-C digestion produced more lysine-containing peptides in almost every molecular weight section (Figure 1(b)). In consideration of undistinguishable arginine-containing peptides produced by trypsin, Lys-C was more suitable for this experiment. We have also compared the Lys-C digestion peptides for human and mouse IPI proteome database. The peptides from two species showed about 30% overlap with each other, which proved the feasibility of our strategy (Figure 1(c)).

### 3.2. Preliminary Experiment Proved the Feasibility of the Design

**Figure 2:**
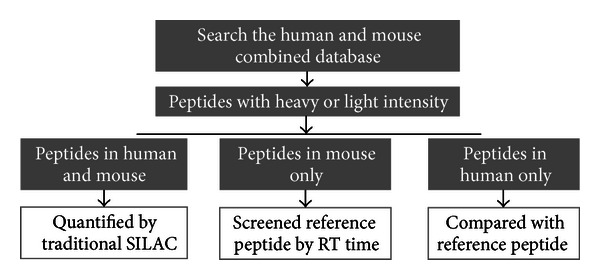
The demonstration for peptide quantification. The peptides were divided into 3 groups. The shared peptides between two species were quantified by traditional SILAC. The mouse-only peptides were taken as reference. And the human-only peptides were compared with co-elution reference peptides to get the quantification results.

Based on the results from theoretic digestions, there were still many human-only peptides. To make better use of these peptides, we divided the quantified peptides into three parts (Figure 2). The shared peptides between human and mouse were quantified by traditional SILAC method. The isotopic-labeled mouse peptides with same sequences were used as internal standards. The mouse-only peptides were screened, and only the peptides found in all of the experiments were kept as references. And then the human-only peptides could be quantified by isotopic-labeled mouse reference peptides with the nearest retention time [18].

**Figure 3:**
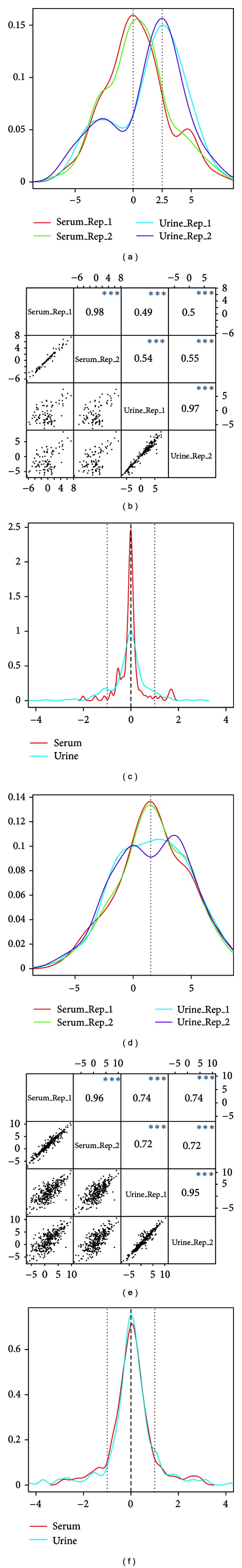
The quantitation results in preliminary experiment. (a) The ratio distribution of shared peptides. (b) The scatter plot and correlation coefficient for quantitation results of shared peptides. (c) The ratio distribution of shared peptides in comparison of replication experiments. (d) The ratio distribution of human-only peptides. (e) The scatter plot and correlation coefficient for quantitation results of human-only peptides. (f) The ratio distribution of human-only peptides in comparison of replication experiments.

To ensure the feasibility of our quantitation strategy, preliminary experiment with human serum and urine samples were performed. Initially, the serum and urine from healthy human subjects were collected and mixed with isotopic-labeled mouse serum, respectively. And two technical replications for each sample were performed. Then, peptide quantification was done as illustrated in Figure 2. The shared peptides were quantified by their own SILAC pair. And the human-only peptides were compared with the mouse only peptides found in all of the 4 experiments. At last, the quantification results from two parts were presented (Figure 3).

For the shared peptides, the ratios distributions (Figure 3(a)) for serum and urine peptides were different. The log transformed serum ratios were around zero, indicating the high consistency between human serum and mouse serum, while the log transformed urine ratios were composed by two peaks above or below zero, indicating that the concentration of shared peptides in human urine was different with mouse serum. These results were reasonable as the mouse serum should be similar with human serum, but different with human urine. We then analyzed the correlation coefficients between 4 experiments (Figure 3(b)). Both the scatter plots at lower side and the correlation coefficients at upper side indicated the high reproducibility between replication experiments, and the disperse points between serum and urine proved the reliability of the results at the other side. The comparison between the results in two replication experiments was also performed (Figure 3(c)). The narrow distribution for the ratios between replication experiments demonstrated that the strategy was applicative in both serum and urine.

The analysis for human-only peptides also showed advantageous result. In ratios distributions, there was a little shift for serum ratios and no obvious peak for urine ratios (Figure 3(d)). The correlation coefficients were a little lower than shared peptides (Figure 3(e)), and the ratios between replication experiments were a little wider but still within twofold changes (Figure 3(f)). These slight changes resulted from the variations introduced by reference peptides with different sequence, which indicated that the quantification for human-only peptides was also reliable.

### 3.3. Application in IgA Nephropathy Samples

IgA nephropathy is the most frequent type of glomerulonephritis and characterized at biopsy by a wide variability of features. Baseline proteinuria is one of the main predictors of IgAN progression. But few studies have evaluated whether some other components of proteinuria could improve the prediction of IgAN and evaluate the effect of therapeutic approaches.

**Figure 4:**
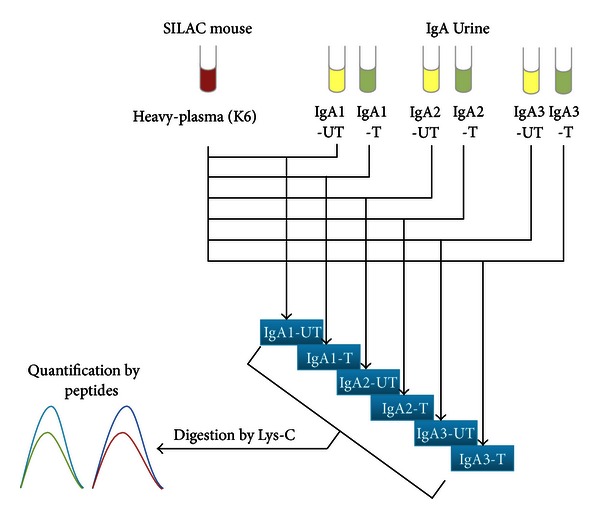
The experiment design for comparison of IgA patients' urine untreated and treated. The UT in the end of sample name indicated that the sample was untreated, and T in the end of sample name indicated that the sample was treated.

Here the novel strategy was applied in the urine samples from IgAN patients treated and untreated. There were totally 3 patients, and two technical replications for each sample were performed (Figure 4).

**Figure 5:**
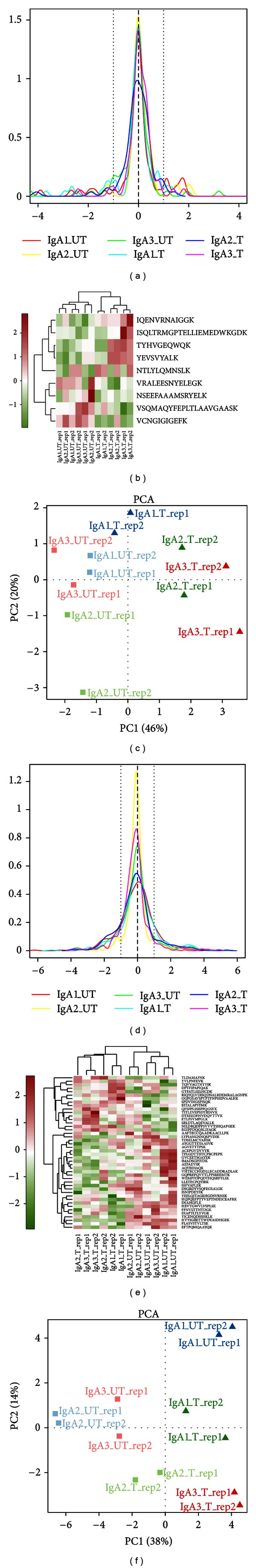
The quantitation results in analysis of IgA patients' urine. (a) The ratio distribution of shared peptides in comparison with replication experiments. (b) The hierarchical cluster analysis result of shared peptides. (c) The principal component analysis result of shared peptides. (d) The ratio distribution of human-only peptides in comparison with replication experiments. (e) The hierarchical cluster analysis result of human-only peptides. (f) The principal component analysis result of human-only peptides.

The quantification results were also from shared peptides and human-only peptides, and the mouse-only peptides found in all samples were used as quantification reference (Figure 5). Firstly, the quantitation ratios between replication experiments for each sample were demonstrated (Figures 5(a) and 5(d)). Most of the ratio changes for replication experiments in each sample were within 2 folds.

Then, the limma package in R environment was used to find significant changed peptides between two groups. As a result, 9 and 44 peptides were selected, respectively, in shared peptides and human-only peptides with *P* value less than 0.05. Hierarchical cluster analysis (HCA) and principal component analysis (PCA) were then performed for the concentrations of these significant peptides, respectively (Figures 5(b)-5(c), and 5(e)-5(f)). In the HCA results, the replicate experiments were first grouped together, and then the samples treated and untreated were separated. In the PCA result, the replicate experiments located together, and the samples treated and untreated were separated by principal component 1. All of the previous results emphasized the high quality of our quantitation data.

### 3.4. Proteins Influenced by the Treatment of IgA Nephropathy

To further analyze the proteins influenced by the treatment and reveal their association with IgA nephropathy, a detailed literature search was performed. And the significant proteins associated with nephropathy were listed in Table 1.

Complement C3 is the most significant decreased protein after treatment in our result. IgA nephropathy is characterized by IgA deposition, and it is frequently accompanied by complement C3. The serum IgA/C3 ratio is taken as a diagnostic marker for the progression of IgAN [29]. And serum C3 was also proved to correlate with prognosis of IgAN [19, 30]. Because of the good correlation between urinary C3 concentrations and the deposition of C3 in glomerular capillary walls, the urinary level of complement C3 was also taken as an accurate indicator of continuing activity of glomerulonephritis [31]. The decrease of complement C3 in our results indicted the curative effect of the treatment.

Albumin is the most famous indicator for renal disease [21, 32, 33]. It is the most abundant protein in disease urine and was clinically used to estimate the progression of renal disease [34]. There were four peptides belonging to albumin that showed significant difference between two groups, which indicated the treatment had effectively influenced the urine proteome.

Besides, we have also found four proteins, which are vitamin D binding protein [23, 35], ceruloplasmin [25], pigment epithelium-derived factor [27], and transferrin [28]. The urinary levels of them were reported to correlate with the development of renal disease. Vitamin D binding protein (VDBP) is responsible for binding to vitamin D and its metabolism products in plasma and then transports them to target tissues. As early as 1977, VDBP was found to be of underexcretion in the plasma of nephropathy patients, but of overexcretion in their urine [36]. The loss of plasma VDBP from urine was taken as the primary reason for vitamin D deficient in nephropathy patients. And the decrease of VDBP after treatment implied its loss was inhibited. Ceruloplasmin (CP) is the major copper-carrying protein in the blood and in addition plays a role in iron metabolism. The urinary copper concentrations were found to significantly increase in macroalbuminuric patients, which might be due to the excretion of CP and damaged renal tubules [25]. And the urinary changes of CP in IgA patients were confirmed by western blot [26]. Besides, the concentration of transferrin in the urine of IgA patients was validated by ELISA experiment [22]. We have also found some other proteins that had not been directly associated with nephropathy, such as insulin-like growth factor-binding protein 7 (IGFBP7) and Apolipoprotein A-I (ApoA1). Some other members of IGFBP family were reported to increase in urine from diabetic nephropathy patients [37], and ApoA1 was found to increase in the plasma of diabetic nephropathy patients [24]. All of these previous reports indicated their potential roles as indicator for nephropathy progression.

On the other side, there were some proteins of overexcretion in the urine of treatment group, including Annexin A1, Cystatin-A, Angiotensinogen, Gamma actin-like protein, and Zinc-alpha-2-glycoprotein. Most of these proteins had never been reported as nephropathy biomarkers or only found to be significant in large-scale expression analysis [38, 39]. As the treatment globally influenced the urinary proteome and the overall protein excretion was reduced in treatment group, the resource and function of overexcretion proteins need more investigation.

## 4. Conclusions

In this work, we proved that SILAC-labeled mouse serum could be used as internal standard for human body fluid proteome analysis. It provided comprehensive and reliable quantitative results and could specifically address the obstacles in this field. With the application of this strategy in patient samples, we can obtain valuable findings in biomarkers discovery for monitoring disease progression and evaluating therapeutic efficacies.

## Figures and Tables

**Table 1:** The significant proteins associated with nephropathy.

Peptide sequence	Protein name	Fold changes treated : untreated	Relevance with nephropathy	Reference
ACEPGVDYVYK	Complement C3	−3.3	Reported as a potentially novel predictor of progressive IgA nephropathy	[19]

HEVTGWVLVSPLSK	Insulin-like growth factor-binding protein 7	−3.2	The urinary levels of other IGFBPs correlated with the development of renal disease	[20]

VHTECCHGDLLECADDRADLAK	Albumin	−2.3	Most famous indicator for nephropathy	[21, 22]
YICENQDSISSKLK		−2.0		
AEFAEVSK		−1.9		
AAFTECCQAADKAACLLPK		−1.6		

HQPQEFPTYVEPTNDEICEAFRK	Vitamin D-binding protein isoform 3	−2.1	Enhanced excretion in urine during diabetic nephropathy	[23]

DSGRDYVSQFEGSALGK	Apolipoprotein A-I	−2.1	Increased in the plasma of diabetic nephropathy patients	[24]

HYYIGIIETTWDYASDHGEK	Ceruloplasmin	−1.7	Enhanced excretion in urine during diabetic nephropathy	[25, 26]

SSFVAPLEK	Pigment epithelium-derived factor	−1.6	A urinary marker for diabetic nephropathy	[27]

DSAHGFLK	Transferrin	−1.6	Enhanced excretion in urine during diabetic nephropathy	[22, 28]
